# Discovery of spermatogenic activators: a lesson from bone morphogenetic protein 8

**DOI:** 10.18632/oncotarget.21597

**Published:** 2017-10-07

**Authors:** Fang-Ju Wu, Ching-Wei Luo

**Affiliations:** Ching-Wei Luo: Department of Life Sciences and Institute of Genome Sciences, National Yang-Ming University, Taipei, Taiwan

**Keywords:** male infertility, spermatogenesis, spermatogenic activator, BMP8, SMAD

Infertility is considered to be the third most serious problem in public health in the 21^st^ century and has affected at least 48.5 million couples worldwide [1]. It is estimated that male factors contribute to 40-50% of all infertility cases [2]. In male infertility, oligozoospermia and azoospermia are two major phenotypes, which can be caused by idiopathic primary testicular dysfunction, chemotherapy, endocrinopathies and other defects that lead to reduction or arrest in spermatogenesis [3]. For some of these patients, application of medical therapies that would induce or assist complete spermatogenesis inside or outside the original testicular environment may help to produce functional sperms suitable for artificial fertilization.

The success in generating functional sperm *ex vivo* reported in 2011 indeed brings hope to these patients [4]. Using this organ culture system, functional spermatids and flagellated sperm can be obtained from cultured neonatal mouse testis explants *in vitro* and the resulting meiotic cells can further give rise to healthy and fertile offspring via microinsemination. This culture method has later been successfully applied to cryopreserved mouse testis tissues [5]. However, while making a breakthrough in production of functional sperm *ex vivo*, the efficiency is no doubt a major concern. The proportion of meiotic cells in cultured testis explants (around 1.71 %) is much lower than that in the mature testis (over 50 %) [4]. Thus, development of extrinsic spermatogenic activators, which can ideally be supplemented in the culture to enhance production of functional sperm, is greatly needed in order to improve the efficiency of spermatogenesis *in vitro*. Such exploitations will not only help characterization of the mechanisms underlying spermatogenesis but also would speed up the clinical application for treating male infertility.

Recently, our data demonstrated that bone morphogenetic protein 8A (BMP8A) can act as a novel spermatogenic activator [6]. As compared to the basal medium, addition of BMP8A protein significantly increased the proportion of meiotic cells in cultured mouse testis explants from two weeks onward. Specifically, almost a seven-fold increase was shown at the third week of culture. In addition to this, the expressional profile and the potential downstreams of BMP8 which lead to the above effect were also characterized. *Bmp8a*, but not *Bmp8b*, was expressed abundantly in the mouse spermatogonia. Unlike most BMPs, BMP8A protein activated not only canonical SMADs 1, 5, and 8 (SMAD1/5/8) signaling but also SMADs 2 and 3 (SMAD2/3) signaling in the mouse spermatogonia. Through signal dissection and receptor characterization, we clarified that the resulting SMAD2/3 signaling, potentially through activation of receptor complexes formed by ALK5 type I receptor and ACVR2A, ACVR2B, or TGFBR2 type II receptor, promoted the proliferation of spermatogonia, whereas the resulting SMAD1/5/8 signaling, potentially through activation of receptor complexes formed by ALK3 type I receptor and ACVR2A or BMPR2 type II receptor, can direct the subsequent differentiation of spermatogonia. Taken as a whole, the dual functions exerted by BMP8A may increase the proportion of differentiating spermatogonia to enter meiosis. Thus, BMP8A can be potentially developed as the first kind of extrinsic spermatogenic activator supplemented in the culture to improve the spermatogenesis efficiency *ex vivo*.

Although supplement with BMP8A significantly increased the final outcome of meiotic cells in cultured testis explants, some concerns should be noted. Firstly, we demonstrated that the BMP8A-induced spermatogonia were tended to differentiate rather than to maintain stemness; this may exhaust the stem cell pool after one round of spermatogenesis. Secondly, BMP8A signaling alone seems not be sufficient for spermatogonia to trigger the initiation of meiosis, as reflected by no change in some meiosis-related gene in treated spermatogonia. This indicates that finding of other spermatogenic activators capable of promoting the completion of diverse stages of spermatogenesis is demanded. Complying with the above concept, several soluble molecules can be considered. For example, colony stimulating factor 1 may be tested to serve as an extrinsic spermatogenic activator that helps expansion of stem cell population in the culture system, as addition of this factor has been shown to enhance the self-renewal of spermatogonial stem cells *in vitro* [7]. Retinoic acid, a key factor required for the initiation of meiosis in the germ cells of both male and female mammals [8], can be selected as one of the activator candidates that promotes meiotic efficiency of developing spermatogonia *in vitro*. In summary, by characterizing the profiles of spermatogenic activators contributing to different stages of spermatogenesis, an efficient cultured procedure using these factors may be developed to recreate limitless cycles of spermatogenesis *in vitro* and thus will benefit future treatment for male infertility.
